# Behavioral Ecology of Captive Species: Using Bibliographic Information to Assess Pet Suitability of Mammal Species

**DOI:** 10.3389/fvets.2016.00035

**Published:** 2016-05-20

**Authors:** Paul Koene, Rudi M. de Mol, Bert Ipema

**Affiliations:** ^1^Animal Welfare, Wageningen Livestock Research, Wageningen University and Research, Wageningen, Netherlands

**Keywords:** behavioral ecology, pet suitability framework, companion animal, pet animal, exotic pet, best professional judgment, behavioral needs, welfare risks

## Abstract

Which mammal species are suitable to be kept as pet? For answering this question many factors have to be considered. Animals have many adaptations to their natural environment in which they have evolved that may cause adaptation problems and/or risks in captivity. Problems may be visible in behavior, welfare, health, and/or human–animal interaction, resulting, for example, in stereotypies, disease, and fear. A framework is developed in which bibliographic information of mammal species from the wild and captive environment is collected and assessed by three teams of animal scientists. Oneliners from literature about behavioral ecology, health, and welfare and human–animal relationship of 90 mammal species are collected by team 1 in a database and strength of behavioral needs and risks is assessed by team 2. Based on summaries of those strengths the suitability of the mammal species is assessed by team 3. Involvement of stakeholders for supplying bibliographic information and assessments was propagated. Combining the individual and subjective assessments of the scientists using statistical methods makes the final assessment of a rank order of suitability as pet of those species less biased and more objective. The framework is dynamic and produces an initial rank ordered list of the pet suitability of 90 mammal species, methods to add new mammal species to the list or remove animals from the list and a method to incorporate stakeholder assessments. A model is developed that allows for provisional classification of pet suitability. Periodical update of the pet suitability framework is expected to produce an updated list with increased reliability and accuracy. Furthermore, the framework could be further developed to assess the pet suitability of additional species of other animal groups, e.g., birds, reptiles, and amphibians.

## Introduction

### Background

Traditional companion animals are more and more replaced by exotic animals (1). The keeping of exotic pets is associated with issues of species conservation, animal and human health, and welfare. For instance, welfare may be compromised because exotic species have often complex needs, such as specific diets and complex social and physical environments. As a consequence, they may show health problems and problem behaviors, such as self-mutilation and stereotypic behaviors. Dogs and cats are often seen as companion animals and hamsters and rabbits as pets, but definitions or use of the terms are largely stakeholder dependent and circular as in: companion animals are animals kept by humans as pets. We define pet and companion animal as synonymous and as an animal that is caged, tamed, exotic, or domesticated and kept as companion for pleasure. Although it should be self-evident that every pet needs a responsible and motivated owner with some knowledge about the animal in his/her care, it is not that self-evident and, therefore, the Dutch Animals Act is implemented to anticipate welfare problems. In the new Animals Act in the Netherlands, which came into effect at January 1, 2013, it is prohibited to keep mammal species as pets. Exceptions are species on the so-called “production animal” list (see Selection of Species to Be Analyzed) and animals on a positive list, i.e., animals that are suitable to be kept as pets by anyone. In the Animal Act, the intrinsic value of animals is recognized, i.e. animals are beings with feelings. Also the five freedoms are applied although in an adapted version, i.e., animals should be free of thirst, hunger and improper diet, of physical and thermal discomfort, of pain, injury and disease, of fear and chronic stress, and of limitations of their natural behavior. Compared to the fourth freedom, i.e., freedom to express normal behavior,^1^ more value is given to the natural behavior of a species. Defined in the Animals Act, nine criteria are given for designating species to a so-called positive list, i.e., animals that are allowed to be kept by an owner without specialist knowledge and skills so without special education or training. Criteria are as follows: (1) the extent to which the animal has to move and needs a specific environment, (2) the average size of the animal in adulthood, (3) the needs of the animal to have periods of activity or inactivity, daily or seasonally, (4) the needs of the animal with respect to forage and food, including composition, (5) the extent to which the animal needs security and shelter, (6) the needs of the animal with respect to reproduction and rearing young, (7) the needs of the animal with regard to cleaning behavior, (8) the social and biosocial needs of the animal, and (9) the extent to which the animal needs incentives and distraction. Furthermore, the species (1) provides no unacceptable degree of hazard to humans or animals, (2) is not prohibited by articles of the Dutch Flora and Fauna Act, and (3) causes no unacceptable harm to the health or welfare of those animals. The prohibitions apply only to mammals. In addition to the Animals Act, the Andibel judgment (Case C-219/07 of 19 June 2008 of the European Court of Justice) is relevant.^2^ It states that the species must be easy to maintain and can fulfill their essential physiological, behavioral, and ecological needs; species may not be aggressive in nature and provide no particular risk for human health; bibliographic data on keeping these animals should be available. Species should not constitute an ecological threat, and in cases of conflicting information the benefit of the doubt should be given to the animal. Important is that the construction of a list of species suitable to be kept as pet (positive list) must be based on objective and non-discriminatory criteria. A procedure should be provided that allows placing new mammal species on such a list. If it proves impossible to determine the risk of keeping a species, it may be justified to apply the precautionary principle and remove it from the list.

### Biology and Needs

The above-mentioned criteria are important factors that could determine placement on such a positive list. Schuppli and Fraser provide a comprehensive description of most relevant issues, i.e., the welfare of the animal, the welfare of the owner, and the welfare of the environment (2). In their framework, natural behaviors or natural behavioral needs have only a limited place; while in the Netherlands, the focus is on the main factors natural behavioral needs and welfare risks in captivity, and the animal is more central than the owner. Currently, the Dutch method is less focused on natural behavioral needs and instead focuses on concrete risks and juridical considerations but this paper focuses on the original approach.

The key starting point for the analysis of the mammal species and their suitability as pets relates to their natural behavior and the associated behavioral needs of the animal. Natural behaviors include some difficulties as they may be unwanted, such as agonistic behaviors. A behavioral need of a species is the need to perform the behavior even if the physiological needs underlying the behavior are satisfied (3). Differences in strength of behavioral needs between species can be illustrated with the example of generalists and specialists. Species are often faced with a choice between performing well on a limited number of activities or behaviors (specialists) or less well on many activities (generalists) (4). Specialists are often adapted to a specific environment, where they have a high fitness. Generalists are “specialized” in order to survive in widely varying environments. Specialists have specific adaptations to their environment, a high behavioral specialization, special behaviors, and probably specific needs. An indication of specialization can be morphology (the study of the shape of animals), think of, e.g., mink with their webbed toes (adapted to a wet environment), the blind mole rat and its intricate social behavior and associated morphology (adapted to a poor desert environment). A flying squirrel has various adjustments, such as a patagium (skin), special muscles, and probably associated brain mechanisms for flying. High behavioral needs in nature may imply that species have a high welfare risk and show abnormal behaviors when in another suboptimal – environment (5). Examples are rooting in pigs (6, 7), feather pecking in hens (8, 9), stereotypies and juvenile mortality in carnivores (10), and stereotypies in gerbils (11, 12).

### Welfare and Risks

Comparison of adaptations in the natural environment and the challenges in a new environment show that natural adaptations may be also functional in the new environment (13). Also, natural adaptations may exist that are not needed anymore, while there are new environmental challenges to which the animal lacks the necessary answers (13). In those last circumstances, unfulfilled motivations may be expressed as abnormal behavior, such as stereotypies. Probably generalists will respond differently to new environmental challenges than specialists (5). Relationships between the quality of the original environment and the behavioral needs of the animal (species) exist and coincide with the occurrence of welfare risks or behavioral problems in the new environment. The more the environment is matched to the behavioral needs of the animal, the less welfare problems are to be expected. Thus, how suitable is an animal for the environment in which it is kept as pet? Is there reason to suppose behavioral, welfare, or health risks? The Schuppli and Fraser framework (2) is applied to parrots (14) and primates (15). These analyses are very general and not species-specific. Furthermore, little emphasis is given to natural behavioral needs and the potential and the actual occurrence of welfare problems. Recently, adaptations to the framework are suggested especially for exotic pets (1). An easy method (16) is developed following Schuppli and Fraser, the first presentation of our method (17) and the method used for creating the Belgian positive list (18). Also the human–animal relationship (HAR) is important but is not often investigated (19, 20). Characteristics of animal species in the wild may be predictive for HARs (5, 10). For instance, a relationship between the minimum home range of carnivores in nature and the amount of stereotypies and mortality of young in captivity is found (10). For determining the pet suitability of a mammal species, the strength of behavioral needs and welfare risks should be part of the framework.

### Assessment of Needs and Risks

In most cases, no data on strength of behavioral needs and welfare risks are available. On base of bibliographic descriptions, experts may be able to estimate/assess behavioral needs, welfare, and health risks of animal species (21–26). However, different opinions and controversies exist concerning pets. For instance, opinions could be that animals choose to live as pets vs. do not choose to be pets, pets are properly cared for vs. are not cared for, pet ownership benefits human health vs. pets are a risk to human health, and humans can do all they want to animals vs. animals should live in their natural environment. Differences in assessments are expected from assessors representing different opinions, experts or stakeholder groups (27–31). Judgments of zoo professionals are often biased because of the relation with the animals under their care and because they may show other behavior in their presence than in their absence (32). Stakeholders may judge animal welfare markedly different from behavior and welfare professionals, as is shown for instance in horse welfare (33). They may also differ in their basic attitudes and judgments of the pet suitability of mammals (34), i.e., some stakeholders state that all mammals are suitable to be kept as pets, others state that most mammals are not suitable without specific skills and knowledge of the owner. Differences are also expected with respect to species similarity to humans (35) and animal use as, for instance, in research (36). In addition, differences in basic attitudes toward animals and animal welfare are also expected in animal scientists (37). Possible basic attitudes are as follows: (1) commercial attitude with focus on activities, such as purchase, sales, marketing products, and services, (2) human-centered attitude with focus on the welfare, education and health of other people, (3) orderly attitude with focus on working with data, records, and systems, (4) natural and environmental attitude with focus on sustainable agriculture, healthy food, and a good living environment for humans, plants, and animals (34). Such differences in basic attitudes may give assessors different views on behavioral needs, welfare risks, and suitability of mammal species as pet. Some additional factors are important for determining suitability, such as danger to the owner and danger to the environment (2). The factor whether the species is domesticated or wild is also very important. For instance, dogs and cats are free roaming in the house, but may show welfare problems when kept in cages (38). In the literature, much more information about behavioral needs and welfare risks is available of domesticated species than of wild species. Based on available data, there is a chance that dogs, cats, rabbits, and guinea pigs are assessed as unsuitable, while wild animals are judged suitable to be kept as pet. The precautionary principle is expected to work in the opposite direction, i.e., preventing that species about which only little information is available are assessed as suitable. Thus, the amount of information available is expected to be a critical factor.

### Scope of This Paper

In this paper, a behavioral ecology approach for keeping animals is taken in parallel to the approach taken toward zoo animals (39). The basis of the system is formed by bibliographical information that by assessments of scientists is converted to strength of the behavioral needs, welfare, health, and HAR risks, both in natural and captive conditions. Expert opinion has been used earlier to address the critical lack of data (40, 41). For a sensitive topic as pet suitability of species with its implications for human individuals and their pets, but also more general for social, economic, and political consequences, conclusions based on expert opinion should be drawn with care for the accuracy of and the differences in opinion concerning data. A group of eight experts is probably adequate because a weighted average of the different opinions of individual experts appeared to be probably accurate and reliable (41). The result of expert opinions and assessments should be a list of relative pet suitabilities of mammal species, based on the combined – subjective – assessments of experts about objective scientific characteristics in relation to their environment. Several methods may be used for this purpose, for instance, a thorough literature review, followed by panel discussion and judgment (42, 43). We chose for a oneliner approach with a oneliner defined as a succinct, meaningful, and accurate statement characterizing a species, linked with a bibliographic source. Allocating oneliners to predefined (sub) criteria and assessing their importance provides the basis for species comparison. Our framework is in agreement with the main lines of the so-called Andibel judgment.^2^ The suitability of mammal species as pets is labeled here *pet suitability*. Application of the approach is expected to produce (1) an initial list that rank orders mammal species concerning pet suitability, (2) suggests methods to add new species to the list or remove animals from the list, (3) emphasizes the role of stakeholders to contribute significantly to the list, and (4) stimulates to incorporate additional factors as domestication and danger to pet owners (zoonosis, accidents) and the environment (invasiveness of species).

## Materials and Methods

### Pet Suitability Framework

**Figure 1:**
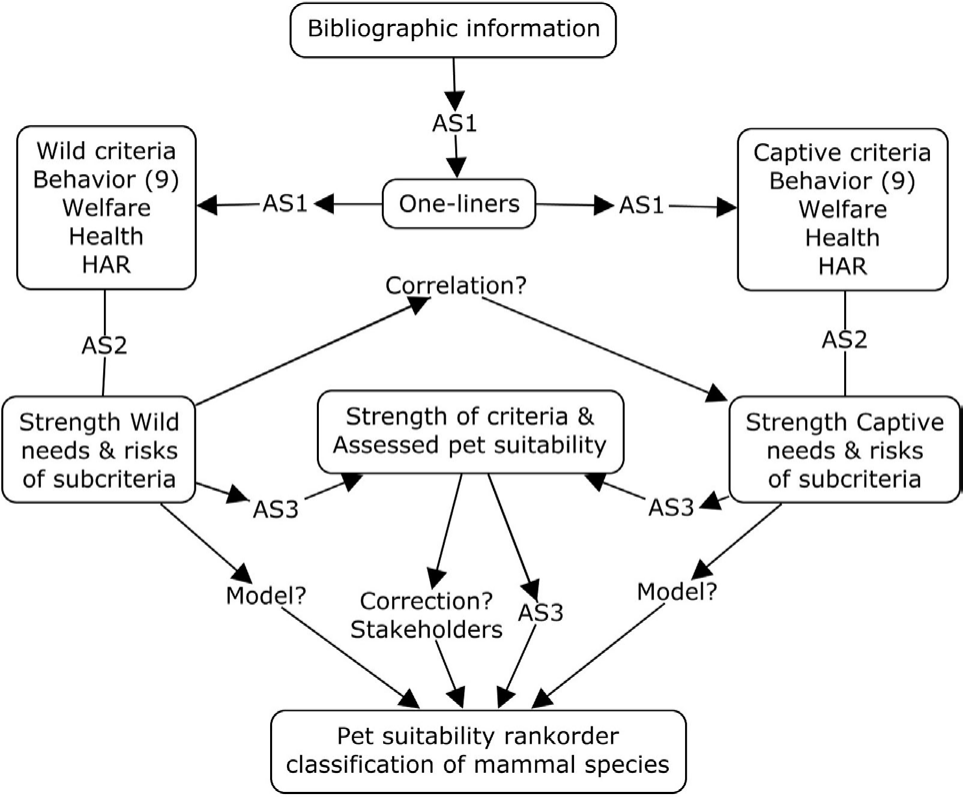
**Framework for analyzing bibliographic information using best professional judgment of animal scientists with an ecology, ethology, or animal husbandry background**. The AS1-team selects oneliners, the AS2-team assesses the strength of needs and risks oneliners and the AS3-team assesses the strength of criteria and the pet suitability. For further explanation see text.

In this section, the step by step process of analyzing bibliographic information is described (Figure 1). Needs and risks are used in this paper as objective and non-discriminatory criteria. Needs and risks are related to behavior, welfare, health, and HAR. Behavior parameters are parameters, mainly indicating needs, while welfare, health, and HAR parameters are parameters, mainly indicating risks (Table 1). In the framework, both types of parameters are used (Figure 1). Hence, the bibliographic material is ordered for the Wild context and for the Captive context (39, 44, 45). The outcome of the process is for each species a place in a rank order, ranging between *unsuitable* and *suitable* to be kept as pet. The process will be explained in the next section.

**Table 1:** **The criteria and subcriteria used for selection of the oneliners of the Wild context and the Captive context to discover behavioral needs and welfare, health and HAR risks**.

Criterion	Label	Description based on needs and/or risks	Sub-criterion labels
1	Space	The space requirements of the animal are estimated on basis of the movements in the space the species makes during its life	Habitat selection, run (between locations), home range, move (on location), disperse, migration, specific behavior, on land – in tree – underground, other
2	Time	The time requirements of the animal species are estimated on the basis of behavioral changes in relation to changes in time and biological rhythms	Activity/inactivity, day, night, dusk active, rhythms in behavior, sleep, rest, hibernation, seasonal, other
3	Metabolism	The metabolic needs of the species are estimated from behavior in relation to foraging, food intake, and food processing	Food items, prey selection, search food, food consumption, hide food, parasitism, drink, urinate, defecate, other
4	Shelter	The hiding needs of the species are estimated on the basis of behavior in relation to enemies, weather changes, etc.	Shelter, make shelter, anti-predator behavior, other
5	Sex	The reproductive needs of the species are estimated on the basis of sexual interactions and parental behavior within the species	Mating system, sexual selection, competition for partners, mate choice, mate guard, sexual dimorphism, parental care, infanticide, nesting behavior, other
6	Care	The body requirements of the animal species are estimated on the basis of behavior in relation to the maintenance of the own body	Cleaning, grooming, care for the exterior, social grooming, allogrooming, thermoregulation (behavior), other
7	Biosocial	The social needs of the species are estimated on the basis of behavior in relation with conspecifics and individuals of other species	Cooperation/altruism, benefits (positive), social organization, social support, social grooming, allogrooming, helpers present, competition, cost (negative), agonistic behavior, rank-order and hierarchy, territoriality, other
8	Info	The information needs of the species are estimated on the basis of behavior in relation to the biotic and abiotic environment	Exploration, play behavior, information seeking, give information (flag, etc.), communication, other
9	Other	Describes those needs, behaviors, and behavior–environment interactions that cannot be assigned to any of the above criteria directly	Behavior without function, difficult to classify behavior, not yet classified, other
10	Welfare	The welfare of a mammalian species depends on many factors. Unfulfilled behavioral needs, abnormal behavior, and stress indicators	Lack of adaptability, climate, stereotypies, problem behavior, other
11	Health	The health is indicated by an active intact body. There are many types of diseases found in mammalian species, all of which may affect the quality of life of a pet and his/her owner	Hygiene, disease, zoonosis 2, mortality, specific problems, other
12	HAR	The human–animal interaction (HAR) is good when man and animal do not disturb each other or exchange diseases	Human environment, special knowledge needed, domestication, danger, zoonosis 1, fauna danger, other

A number of teams of scientists of the Animal Sciences Group of Wageningen University and Research center was hired and complemented with experts from the Environmental Sciences Group and the Veterinary Faculty of Utrecht University, the Netherlands. The AS1-team [animal (A) scientist (S) team 1; 7 experts – two PhD – are from animal husbandry, ecology, ethology, veterinary sciences] collected and selected oneliners from bibliographic sources. The AS2-team (eight members; four overlap with the AS1-team; PhD or equal, ecology, ethology, veterinary sciences) assessed strength of needs and risks. The AS3-team (eight members; seven overlap with the AS2-team; PhD or equal, ecology, ethology, veterinary sciences) were instructed and assessed data per criterion according to pre-set rules. Despite the instructions, scientists may show differences in their assessments, as they may change with time (tiredness), or may unintentionally be related to size, color, cuddliness, or ugliness of the assessed species. Differences in the assessments of pet suitability between and within scientists and between and within mammal species are corrected using statistical methods (Figure 1). It is also hypothesized that correlations among/between criteria and correlations between criteria and the final suitability facilitates the modeling of the process of assessing pet suitability of animals (Figure 1).

### Selection of Species to Be Analyzed

To gather factual information about the mammal species that are kept privately as companion mammals in the Netherlands, a survey over the internet was opened from June 2012 until August 2012. In the survey, information was requested about pre-selected species but people could also provide information on new species. The data acquired by the survey (#1) are supplemented with bibliographic information on recent numbers of species rescue requests from the (#2) AAP foundation (www.aap.nl; data of 2010), (#3) animal confiscation, the (#4) number of vet visits per species, (#5) animals in petting zoos, (#6) animal shelters and rescues, (#7) animals sold (46), and (#8) animals kept by the members of the Association of Park Animal Owners who keep mainly exotic species. Subsequently, it is investigated in how many of these eight situations, a species is mentioned. For example the degu (*Octodon degus*) is mentioned in all eight situations. Based on the data, it was decided that species at least found in two of the eight contexts were to be analyzed first. In a later stage, all species present in the Netherlands were planned to be analyzed. Rankings for each situation are made based on species and the number present. Species are prioritized based on the rankings. As production animals are allowed to be kept also as pet, the species on the production animal list were not analyzed. “Production animal” list mammals in the Netherlands are: rabbit (*Oryctolagus cuniculus*), brown rat (*Rattus norvegicus*), domestic mouse (*Mus musculus*), Guinea pig (*Cavia porcellus*), golden hamster (*Mesocricetus auratus*), gerbil (*Meriones unguiculatus*), mink (*Mustela vison*), horse (*Equus caballus*), donkey (*Equus asinus*), pig (*Sus scrofa*), goat (*Capra hircus*), cattle (*Bos taurus*), water buffalo (*Bubalus bubalis*), fallow deer (*Cervus dama dama*), red deer (*Cervus elaphus*), and sheep (*Ovis aries*). In addition, the dog (*Canis lupus familiaris*) and cat (*Felis catus*) are not analyzed, because of their way of housing (free roaming), of variation in breeds, the vast amount of literature and of the delicacy of the subject. When the framework is further evolved the aforementioned species should be analyzed. A number of species are not allowed to be kept on the basis of species protection legislation in the Netherlands (i.e., the Flora and Fauna law). The final list included domesticated species and semi-domesticated species.^3^ The remaining species are ordered according to their ranking score, which was determined on presence in the eight contexts and the numbers kept. The final list consisted of 90 mammal species (Table 2).

**Table 2:** **List of mammal species that are analyzed**.

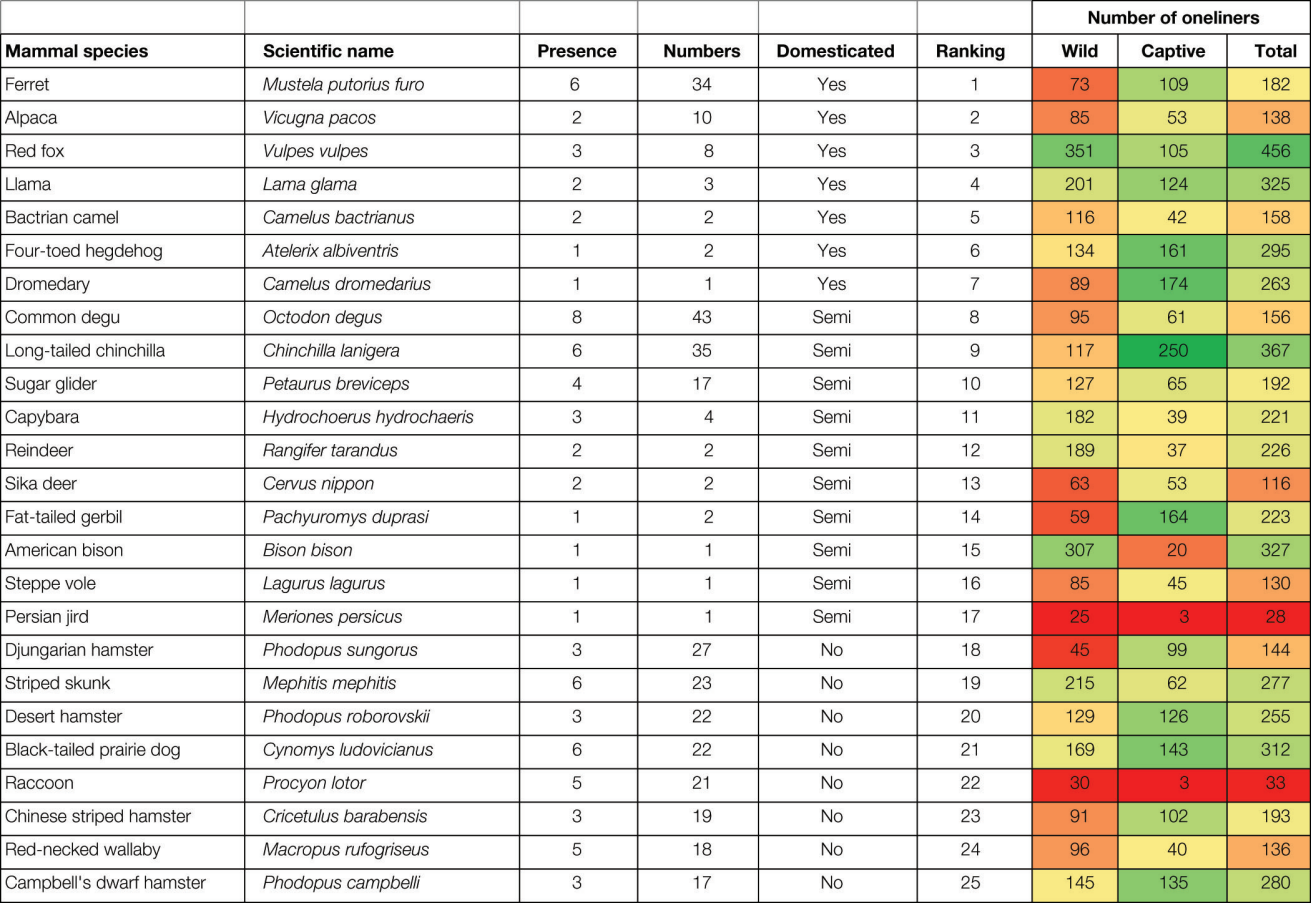

*The first 25 species are shown; the complete list is in Supplementary Material (Data Sheet 1.ZIP). Presence in situations (max. 8), number present in those situations (Numbers), Domesticated according to domestication list (Wikipedia, 2012)^9^ determined the final ranking of species for analysis. Ninety species were selected omitting the production animals, dog, cat, and endangered endemic species. The number of oneliners from the Wild context (Wild), the Captive context (Captive), and the total number of relevant oneliners found (Total) are shown. Green indicates a number of collected oneliners above average and red indicates under average (graded color scale)*.

### The Database

A Microsoft Access database was designed to store all data of species in a structured way according to the framework in Figure 1. The database contains information on behavior, welfare, health, and HARs. The database is structured so that it consists of two symmetrical sections on literature of the species as a wild animal (Wild context) and on literature of the species as a pet (Captive context). Each part consists of 12 criteria, i.e., 9 behavioral criteria (1–9), welfare (10), health (11), and HAR (12). Consequently, there are 12 criteria for animals in the wild (numbered 1–12) and 12 criteria for animals in captivity (numbered 13–24). Both groups of 12 criteria are subdivided into a total of 84 subcriteria (Table 1). This structure is open for future additions, i.e., more subcriteria can be added, for instance, many items described in the Schuppli and Fraser paper (2) can be added to the HAR criterion.

### Bibliographic Information

Literature is collected from various sources and compiled in the database. Web of Science is used as first and main information source (mainly using EndNote as reference database). The search started with the species name in Latin, Dutch, and English. Sometimes species in a genus are found to be quite similar; in that case also, the genus is used in the search but finally all information found is linked to a species. Two basic profiles are applied, of which the one resulting in most references found is used:

- Profile1 is [(zoo or laborat* or companion or pet or pets or home or human or capt* or exotic or invasive or husbandry or management or wild* or natur* or environment*) and (behavior* or enrich* or welfare or well-being or health or domesti* or adapt* or* prefer* or stereotyp* or disease or abnorm*)], and
- Profile2 [(zoo or laborat* or companion or pet or pets or home or capt* or exotic or invasive or husbandry or management or wild* or natur* or environment*) and (behavior* or enrich* or welfare or well-being or health or domesti* or adapt* or* prefer* or stereotyp* or disease or abnorm* or devia* or* or space or time or metabol* or saf* or* sex* or groom or soci* or informat* or human)].

The search was subsequently refined by selection of references from zoology, behavioral sciences, ecology, veterinary sciences, biology, physiology, and environmental sciences. References from neurological and genetic studies are often removed since they resulted in too many references that were not related to a sub-criterion.

Many basic characteristics of species were not easily found in scientific literature. So, additional information is found in – often Dutch – encyclopedias, handbooks, websites, and reports (51–71).^4^^,^^5^^,^^6^^,^^7^^,^^8^^,^^9^^,^^10^ Gray literature is generally defined as academic literature that is not formally published. Gray literature that was delivered in the internet survey or otherwise supplied by stakeholders is also included. Unfortunately, due to limited participation of stakeholders the amount thereof was limited.

#### Oneliners

Findings or one-line quotes (oneliners) are as short as possible verbatim quotes about a characteristic of a species that fitted in one of the 84 subcriteria of the Wild and Captive context and their 12 criteria (Table 1). Only the labels of the subcriteria are given as most speak for themselves. However, some need additional explanation. *Allogroom* appears twice as part of Care and Biosocial, i.e., the oneliner about allogrooming can be put in one or in both criteria dependent on the content and context. *Zoonosis 1* is defined as bringing a disease from non-human animal to humans, while *zoonosis 2* is bringing disease from humans to the animal. *Human environment* deals with the animal in relation to the human environment, especially indicating how well the species can adapt to the human environment. *Special knowledge* indicates whether special knowledge is needed for taking care of the species. *Domestication* indicates how long the species co-exists closely with human beings. *Danger* deals with the question how dangerous the species is for humans. *Fauna danger* indicates danger for the local fauna as invasive species. Preferably oneliners were selected that included an indication of the strength and importance of that trait. For example, this species is *only* a nocturnal animal, this species spent *a rather long time* to eat, it is *essential* that this species has a *high* roost, this species is *not* aggressive toward conspecifics, individuals of the species *cannot* live outside of a group, keeping in small room has *only small* welfare risks, *few* stereotypies result, pellets *often* give oral problems, etc. However, in most oneliners such indications were not found. If a oneliner contains information about more than one sub–criterion, it is assigned to both relevant subcriteria. Findings may, therefore, occur multiple times in the database. The oneliner quotes are distilled from the scientific and gray literature by the AS1-team and every oneliner was linked to its exact bibliographic source. The group was trained by the first author using a PowerPoint presentation of criteria and subcriteria and subsequent discussion of examples and difficult cases. A manual with the following items was used: (1) main focus is to extract information on behavior and welfare of a species as pet, (2) information of the animal in the wild is additional information, (3) on average 4 h were available to extract oneliners from the bibliographic source per species. Each of the trained scientists focused on a limited number of species under supervision of the first author. In case of doubt, a second assessor from the AS1-team was asked to help and double check the selected oneliners for inclusion in the subcriteria.

### Assessment of Oneliners

After finishing the collection of oneliners for all 90 species, they were evaluated and assessed by eight animal scientists (AS2-team). We considered blinding assessors in this and subsequent stages from the name and photo of the species. However, then editing oneliners was needed, the motivation of the assessors was probably lowered and participation of stakeholders reduced. Hence, always explicit species information was available. These assessors were given the task to provide the oneliners of a 1–5 Likert score, i.e., low (1), low-medium (2), medium (3), medium-high (4), and high (5), related to the strength of behavioral needs and/or strength of the welfare risks. More precisely, the estimates were answers to eight questions that differed between the (groups of) items, depending on the parent criterion (Table 3). The importance of a oneliner can often be inferred from the occurrence of behaviors that are consistently present in different conditions and are occurring in the majority of individuals of a species. Thus, natural behavioral needs of a species may be estimated on the basis of literature on behavior in the wild and in captivity. Restriction of behavioral needs – when normal behavior cannot be performed – is supposed to contain a risk of developing abnormal behavior (stereotypies, aggression, apathy, mutilations, etc.). In case of lack of knowledge about the consequences of not being able to perform a behavior, an assessors’ estimate can be based on analogy with other species. Answers with high scores on all questions are assumed to be related with high behavioral needs (possibly related to more behavioral problems), higher welfare and health risks and more HAR problems.

**Table 3:** **Questions asked to the selected animal scientists (AS2, AS3, AS4) and stakeholders for the subcriteria in the database (from low to high needs, from low to high risks)**.

Question	Environment	Crit. nr	Criterion	Given the data type, context, subcriterion, and this finding, what is your answer to the following question?
1	Wild	1–9	Behavior	What is your estimate of the behavioral needs of the species in its wild/natural environment?
2	Wild	10	Welfare	What is your estimation of welfare risks or problems in the wild/natural environment?
3	Wild	11	Health	What is your estimate of health risks or problems in the wild/natural environment?
4	Wild	12	HAR	What is your estimation of problems or risks for human–animal relationship in the wild/natural environment?
5	Captive	21–29	Behavior	What is your estimation of behavioral constraints or problems in the captive environment?
6	Captive	30	Welfare	What is your estimation of welfare problems or risks in the captive environment?
7	Captive	31	Health	What is your estimate of health problems or risks in the captive environment?
8	Captive	32	HAR	What is your estimation of problems or risks for human–animal relationship in the captive environment?

### Assessment of Suitability

The lowest, highest, and average score per sub-criterion were calculated based on assessments of the AS2-team. All scores were graphically presented to scientists and stakeholders for assessment of the strength/importance of a criterion. In the presentation of the data, information is given about the species (scientific and Dutch name), a picture of the species, the total number of oneliners collected per species, the criterion (1 out of 12 per context), the context (Wild/Captive), the number of oneliners per criterion, the average of the scores per criterion, and the question that is asked for the specific criterion (Table 3). More detailed information was made available to the assessor after pressing a button. In a help screen, examples of oneliners were displayed from the subcriteria with the specified average score and the score range. Per criterion two answers were requested, i.e., the strength of the criterion (1 = low, 2 = low/medium, 3 = medium, 4 = medium/high, 5 = high) and whether based on the presented criterion alone the species was unsuitable or suitable to be kept by an owner without special skills and knowledge (*do not know* was the additional option). In case no data were available over all subcriteria within a criterion, the answer to the question was set to missing. The species were presented in alphabetical order. After giving an answer, the next criterion/screen was presented. The assessor was, however, free to start or stop anywhere in the database, i.e., no formal randomization was applied and order effects could not be ruled out. When all 24 criteria of a species had been viewed and scored, a summary screen was presented, in which the preceding scores of the scientist were shown per criterion and context (Wild/Captive), the criterion (1–12, 13–24), the score per criterion (low to high) and the assessed suitabilities based on one criterion. Colors indicated low (yellow) and high (orange) scores and unsuitable (red) vs. suitable (green) assessments. The scientist was asked to give an overall assessment of the suitability of the species as pet (unsuitable, do not know, suitable). Once all questions about a species were answered, the assessor could proceed to the next species in the database. In this way, the scores of needs and risks could be related to the assessed suitability, allowing for formal statistical analysis.

#### Instructed Assessors

We wanted to relate pet suitability of a species to the strength of behavioral needs and welfare risks extracted from bibliographic information. Therefore explicit instructions to the members of the AS3-team were given as follows: (1) natural behavioral needs should be fulfilled as much as possible, (2) welfare risks should be low, (3) the animal keeper is defined as in the Animals Act and is a person “without specialist knowledge and skills,” (4) each species is viewed and judged using the same approach, (5) in case of insufficient or lacking information about a criterion, the assessment is “do not know,” i.e., applying the precautionary principle, (6) consequently, a certain level of lack of information should lead to a final judgment in the 25th screen “unsuitable,” (7) in addition to the strength of needs and risks systematic but reserved application of own criteria is allowed (for instance, related to size, degree of domestication, exotic degree, relative importance of sub–criteria, and criteria themselves, the amount of information), and finally (8) first scroll through the complete database before determining your own individual criteria and answering the questions for all 90 mammal species. Based on this approach, we expected to find significant relations between the data provided and the final assessments of suitability for each of the AS3-team members. The pet suitability assessment of the AS3-team is, for this study, the gold standard and best professional judgment.

#### Uninstructed Assessors

The framework was designed so that stakeholders could provide oneliners, score needs and risks based on the oneliners, and assess pet suitability in the same way as the AS2- and AS3-teams. In this way, each stakeholder could provide a final rank order and classification of species and show their approach of pet suitability. Furthermore, it could show deviations from the scores of the AS3-team, where discussion or dialog was needed and where final assessment of suitabilities could be adapted. For this statistical approach, stakeholders^11^ were requested to provide names of 10–20 representatives who were willing to assess the suitability of the mammal’s species as pets based on the final database. All stakeholders asked by the Dutch government agreed to this.

To investigate the effect of instruction concerning the relation between pet suitability and needs and risks, an additional group of animal scientists (AS4-team; 10 members; PhD or equal, ecology, ethology, veterinary sciences; no overlap with members of other teams) was asked to assess the suitability of species as did the AS3-team, but they were not instructed. Being not instructed, they could provide information that might be comparable to information obtained from – also uninstructed – stakeholders. Finally, the assessments of the AS4-team are explicitly not intended to be used for the final ranking of mammal species suitability and are used only for comparison between instructed and uninstructed assessors and as example of stakeholder assessments.

### Analyses

This framework produced a database with oneliners from the literature (by the AS1-team). Strengths of behavioral needs or risks from oneliners were scored on a scale of 1–5 (by the AS2-team). Data from such Likert scales can be analyzed with parametric statistics (72). On the basis of averages of the scores per criterion per species, the suitability to keep a species as pet was assessed by instructed (the AS3-team) and uninstructed assessors (stakeholders and the AS4-team).

#### Statistical Analyses

In some subcriteria, the collected information was almost identical. For instance, some encyclopedias provided identical information. For time-saving considerations, a maximum of five oneliners per subcriterion per species was analyzed; they were randomly selected. The correlation between assessments of members within the AS2- and the AS3-teams was calculated by Spearman’s Rho (73). The correlation between their criteria scores from the Wild context and those from the Captive context was also calculated using Spearman’s Rho.

The relation between the strength of criteria and the assessed suitability in the summary table is analyzed (1) to verify whether instructing assessors was successful and (2) to statistically eliminate judgment bias between assessors and species. In a multinomial logistic regression (MLR) (73), the data presented in the summary table were analyzed. The dependent variable was the final suitability assessment (suitable, unsuitable, do not know) while the factor was assessor and the co-variates were per species the total low needs (score 1 and 2) in the wild (labeled as Wild Low), high needs (score 4 and 5) in the wild (Wild High), low needs (score 1 and 2) in captivity (Captive Low), and high needs (score 4 and 5) in captivity (Captive High), the total number of criteria assessed *unsuitable* in the wild (Wild Unsuit), *suitable* in the wild (Wild Suit), *unsuitable* in captivity (Captive Unsuit), and *suitable* in captivity (Captive Suit). The medium needs (Wild Mid and Captive Mid) and the missing suitability assessment variables (Wild Missing and Captive Missing) are omitted from the analysis to avoid matrix singularity. The MLR procedure exports per assessor and species the probability of being suitable, unsuitable and do not know, corrected for assessor and species differences. The probabilities of being suitable and unsuitable are integrated in the odds ratio (usually abbreviated “OR”) of pet suitability. The OR quantifies how suitability or unsuitability is associated with the presence or absence of behavioral needs and welfare, health, and HAR risks, in short the criteria. While the OR is not normally distributed, the natural logarithm of the OR, the LOR is normally distributed. Subsequently, the log odds ratio (LOR) is calculated as the Natural logarithm of the odds that the species is estimated suitable, i.e., P_suitable_/(1 – P_suitable_) divided by the odds that the species is estimated unsuitable as pet, i.e., P_unsuitable_/(1 – P_unsuitable_). The LOR_cor_ of the assessors are averaged per species and rank ordered. Species that have a LOR above 0.0 have higher odds of being suitable than being unsuitable. The LOR of the raw assessment is labeled LOR_raw_, while the LOR statistically corrected for assessor and species differences by MLR is labeled the LOR_cor_. This last value is the best estimate of the pet suitability of a species. Differences between the range of LOR_cor_ of the AS3-team and the AS4-team are analyzed using the Moses test of extreme reactions (73).

#### Modeling

Accuracy and efficiency (in time) of the framework may be increased if classifications of suitability of a species could be made in an early stage. Classification of the pet suitability of a species on basis of the current dataset can be done in several ways. It is investigated whether accurate classification could be made from the criteria scores alone using the scores of the AS2-team only and leaving out the time-consuming assessment of the AS3-team. It is expected that the averages of subcriteria per species (AS2-team) are related to the final assessments and corrected probabilities of suitability, unsuitability and thus the log odds ratio of pet suitability (LOR_cor_) of the AS3-team. In case the relation between criteria strength and suitability estimate is strong, new species can be added and rank ordered without using suitability assessments of the AS3-team. In that case, only one group of assessors is needed to determine the suitability of a new mammal species, reducing time spent and costs. The relation between criterion scores and the assessed suitability is analyzed using Automatic Linear Modeling (ALM) (73). ALM is a linear regression procedure that prepares data (transformation, grouping), and handles outliers and missing data according to initial restrictions set by the user to increase predictive power. The regression is done using parameters of Wild criteria alone, of Captive criteria alone and of the combination of Wild and Captive criteria in an ALM regression. The found models are exported (using Export Model), and the LOR_cor_ is estimated by importing the found ALM model in the Scoring Wizard (one of the Utilities of SPSS). Furthermore, a cross-validation is done based on the Wild and Captive criteria and leaving out the suitability estimates of the target species one by one. Leave-one-out cross-validation (LooCV) involves using one species as the validation set and the remaining species as the training set. This is repeated for each species (*N* = 90). For classification of the LOR_cor_ of suitability, the best fit with the criteria is chosen from one of five possible choices, i.e., the average, the weighted average, the minimum, the maximum of the subcriteria, and the number of subcriteria above 3. Correlations between the corrected LOR_cor_ (our standard) and the estimated LORs give an indication of the accuracy of the rank order of suitabilities. The average of the estimates is calculated as the absolute difference between LOR_cor_ and the estimated LOR.

## Results

### Collected Oneliners

**Figure 2:**
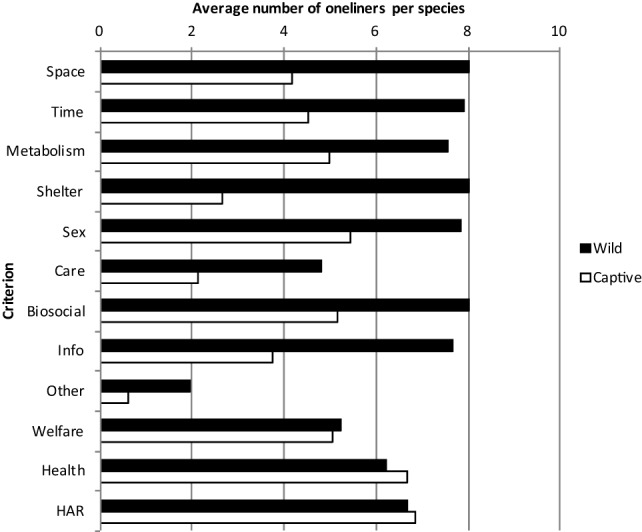
**Average number of oneliners per species from the Wild and the Captive context**. Variation is large and described in the text.

The analysis of the internet inquiry combined with data from reports led to the selection of 90 mammal species. The list with scientific and English names is given in Table 2 (the full list in Supplementary Material). The literature analysis by the AS1-team produced 15,847 relevant oneliners, averaging about 176 oneliners per species. There are large differences between species in the number of oneliners found, and also in the distribution of the findings over the criteria and the Wild and Captive context (Figure 2). For example, the brown bear (*Ursus arctos*) produced most oneliners in the Wild context and the chinchilla (*Chinchilla lanigera*) most in the Captive context. Of many squirrel species, few oneliners were found. Overall, the information that was found came mainly from the Wild context (Table 2, 12,419 oneliners). Concerning the criteria, most information is found for the criteria Biosocial, Space, Sex, and Metabolism in the Wild context (Figure 2). The least is found on the criterion Care.

### Strength of Needs and Risks

**Figure 3:**
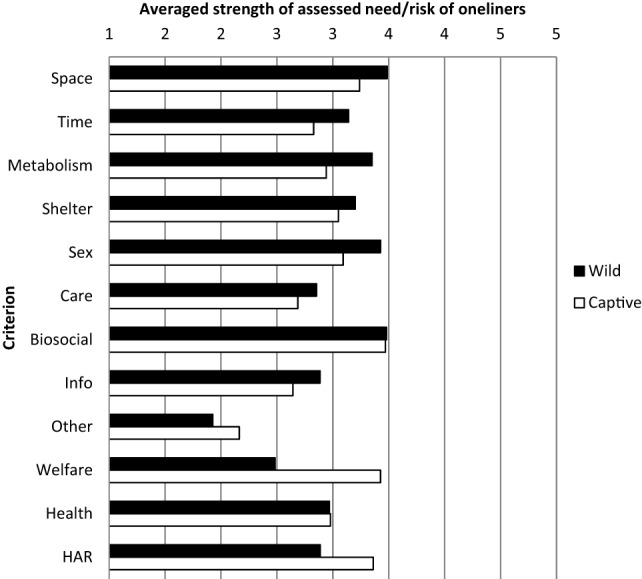
**Average strengths of the 12 criteria from the Wild context and the 12 criteria from the Captive context on a scale of 1–5 by the AS2-team**.

The AS2-team assessed the strength of needs and risks of the oneliners for use in the database. The members of the team received a manual with instructions and suggestions about possible scores/ratings of needs and risks. The scores of the eight members of the AS2-team were averaged per sub-criterion in the database, followed by averaging the estimated strength of the subcriteria per criterion for all species (Figure 3). So, the strength of natural behavioral needs and welfare, health, and HAR risks of the criteria is calculated based on the estimated scores of the oneliners. The criterion Space (see for subcriteria Table 1) is generally the highest rated, followed by Biosocial behavior. Large differences between species with regard to the average over all criteria are found, ranging from 2.17 to 3.71. The correlation between the average scores of criteria of the mammal species (*N* = 90) shows that the correlation between AS2-team members is high indicating that the rank order of the species for each assessor is very similar (above diagonal Table 4). The correlation between the average scores of criteria (*N* = 24) is mostly significant (under diagonal Table 4), indicating that also the rank order of strength of criteria is related between the AS2-team members. Still, individual variation exists and exceptions are the lower correlations with some of the others of assessor 4 and 8.

**Table 4:** Spearman rank correlations (significant Rho = bold, italic; *P < 0.05; **P < 0.01) between AS2-team assessors for average rating of *N* = 90 mammals (above diagonal) and average rating of *N* = 24 criteria (under diagonal).

AS2-team	1	2	3	4	5	6	7	8
1		0.748**	0.539**	0.517**	0.516**	0.645**	0.432**	0.516**
2	0.517**		0.575**	0.433**	0.569**	0.727**	0.518**	0.540**
3	0.430*	0.761**		0.383**	0.373**	0.457**	0.516**	0.418**
4	0.530**	0.230	0.082		0.450**	0.397**	−0.041	0.449**
5	0.330	0.508*	0.733**	−0.217		0.483**	0.136	0.515**
6	0.768**	0.493*	0.405*	0.239	0.459*		0.324**	0.490**
7	0.670**	0.521**	0.632**	0.135	0.581**	0.624**		0.146
8	0.572**	0.457*	0.547**	0.353	0.303	0.376	0.637**	

### Instructed Assessment

Averaged criterion scores (*N* = 24) of AS3-team members were calculated and pairwise correlation between assessors for the mammals species was calculated (*N* = 90). The correlations between AS3-team members were in all cases highly significant (*P* < 0.0001; even higher than those of the AS2-team, Table 4), suggesting that all assessors produced on average criterion scores resulting in roughly the same rank order of species (range of correlations 0.361–0.939, *N* = 90) and criteria (range of correlations 0.419–0.971, *N* = 24).

A high number of criteria from the Wild context correlate with criteria from the Captive context (67 of the possible 144 correlations are significant). As an example, the criterion Welfare in the Captive context correlates significantly (*P* < 0.01) with the behavior criteria from the Wild context, i.e., Space (*R* = 0.192), Time (*R* = 0.429), Shelter (*R* = 0.207), Sex (*R* = 0.290), Care (*R* = 0.296), Biosocial (*R* = 0.183), and Info (*R* = 0.267).

The raw suitability assessment (LOR_raw_) is determined by the odds of the proportion of AS3 assessors, that scored a species suitable and the odds of the proportion that scored a species unsuitable (Table 5). An example of a species that is suitable (six out of eight assessors and LOR_raw_ = 2.20) according to the majority of assessors is the llama (*Lama glama*). An example of a species that is judged by the majority of assessors *unsuitable* (8 out of 8, LOR_raw_ = undetermined) is the black-tailed prairie dog (*Cynomys ludovicianus*). Four assessors found Campbell’s dwarf hamster (*Phodopus campbelli*) *suitable* while four assessors assessed the hamster *unsuitable* (LOR_raw_ = 0.00). No single species is found suitable by all AS3 assessors on the basis of the information given. Based on the raw scores of suitability and unsuitability eight species were estimated suitable (LOR_raw_ > 0) to be kept as pet (*Macropus agilis, Lama glama, Cervus nippon, Paradoxurus hermaphroditus, Galea musteloides, Macropus eugenii, Acomys dimidiatus*, *and Cricetulus barabensis*; Table 5). To fulfill the criteria of the Andibel judgment (objective and non-discriminatory), the differences within and between assessors and species are estimated using MLR (likelihood ratio test, chi-square = 567.56, DF = 30, *P* < 0.001). Assessments differed between assessors (chi-square = 205.22, DF = 14, *P* < 0.001). The LOR_cor_ per species is determined and the 90 investigated species are rank ordered consequently (Table 5). The LOR_cor_ of five species was above zero and had higher odds to be suitable as pet (*Cervus nippon, Macropus agilis, Macropus eugenii, Lama glama*, and *Paradoxurus hermaphroditus*). Thus, correction of the assessments of the AS3-team removed *Galea musteloides*, *Acomys dimidiatus*, and *Cricetulus barabensis* off their list.

**Table 5:** **Logarithmic odds ratio of pet suitability (assessed and corrected using Multinomial Logistic Regression of indicators of Wild and Captive criteria; see text)**.

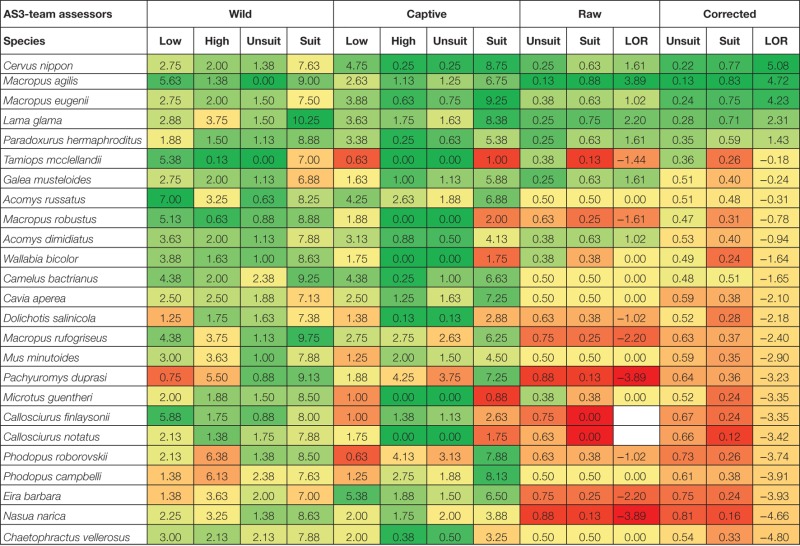

*The first 25 species are shown; the complete list in Supplementary Material (Data Sheet 1.ZIP). Colors are based on conditional formatting in MS Excel 2010. All cells are formatted per column based on a three- color gradual scale from green (related to a high suitability as pet), via yellow (related to a medium suitability as pet) to red (related to a low suitable as pet). Empty cells could not be estimated*.

### Uninstructed Assessment

#### Stakeholders

The number of members of stakeholder groups that finally participated as assessors is relatively low and the results cannot be interpreted as reflecting the views of those involved in the debate on the pet suitability of mammal species in the Netherlands (discussions on the so-called Positive List). Of the selected stakeholders only some representatives of animal welfare NGOs responded positively to participate, each stakeholder (NGO1 and NGO2) had two assessors; the analysis was also done combining both stakeholders (NGO). The raw scores of the NGO1-team revealed no species with a pet suitability (LOR_raw_) above zero. The NGO2-team estimated five species suitable as pet (*Cavia aperea, Mustela putorius furo, Phodopus roborovskii, Phodopus campbelli*, and *Phodopus sungorus*) and both NGOs combined showed one species with a LOR_raw_ above 0 (*Phodopus roborovskii*). Corrected pet suitabilities for the NGO-teams are estimated by MLR (likelihood ratio test, chi-square = 143.73, DF = 22, *P* = 0.000). Only NGO2 showed species with a LOR_cor_ above 0 (*Mustela putorius furo, Phodopus campbelli, Macropus eugenii, Vicugna pacos, Lama glama, Phodopus roborovskii, Cavia aperea, Camelus dromedarius, Vulpes lagopus*, and *Phodopus sungorus*). Most of them are (semi-)domesticated species. Thus, correction of the raw NGO2 assessments adds *Macropus eugenii, Vicugna pacos, Lama glama, Camelus dromedarius*, and *Vulpes lagopus* to their list.

#### Animal Scientists

Additionally, 10 animal researchers were asked to fill in the database questions without the specific instructions given to the instructed assessors (the AS4-team; no overlap with the AS3-team; see Instructed Assessment) to find out the effect of instruction and to simulate stakeholder assessments. The raw and corrected scores of the AS4-team indicated no species suitable as pet. The range of assessments of the AS4-team was significantly different from that of the AS3-team (Moses test of extreme reactions = 10, *P* = 0.008). Five researchers of the AS4-team found no species suitable as pet. The other five researchers assessed on average 11 species suitable as pets (LOR_raw_ > 0; *Cervus nippon, Lama glama, Paradoxurus hermaphroditus, Acomys russatus, Cricetulus barabensis, Macropus rufogriseus, Vicugna pacos, Camelus bactrianus, Galea musteloides, Callosciurus finlaysonii*, and *Dolichotis patagonum*). Based on the above difference the group of 10 uninstructed assessors is split into an AS4-high and an AS4-low group as examples of groups that assessed more species or less species suitable compared to the AS3-team. Corrected pet suitabilities (LOR_cor_) of the AS4-high team are estimated by MLR (likelihood ratio test, chi-square = 620.57, df = 34, *P* < 0.001) and 12 species showed above zero pet suitabilities (*Macropus agilis, Cervus nippon, Lama glama, Macropus eugenii, Macropus rufogriseus, Paradoxurus hermaphroditus, Acomys russatus, Camelus bactrianus, Macropus robustus, Cavia aperea, Tamiops mcclellandii*, and *Vicugna pacos*). Correction of the AS4-high team puts *Macropus agilis, Macropus eugenii, Macropus robustus, Cavia aperea*, and *Tamiops mcclellandii* on their list and removes *Galea musteloides, Callosciurus finlaysonii, Cricetulus barabensis*, and *Dolichotis patagonum* off their list.

### Model of Suitability Based on Oneliner Strengths

The relation between LOR_cor_ and criteria averages is explored with an ALM (73) using initially five parameters of the subcriteria, i.e., the average, the minimal, the maximal, the weighted average, and the selection of the subcriteria with values above 3. The following explained variances were found for the five parameters: (1) averaged subcriteria/oneliner assessment (79.9%), (2) weighted average oneliners (82.0%), (3) maximum subcriteria (75.9%), (4) minimum subcriteria (55.0%), and percentage above 3 (74.7%). We analyzed the relationship using the weighted average of the criteria assessed by the AS2-team. LOR_cor_ of species were estimated on base of Wild criteria, Captive criteria, and the combination of Wild and Captive criteria (Table 6). Cross-validation leaving the target species – one species – out (LooCV) resulted in an estimated LOR_cor_ for each of the 90 species. Correlations of the standard LOR_cor_ with the estimated LOR_cor_ were for Wild criteria (*R* = 0.768), Captive criteria (*R* = 0.800), Wild and Captive criteria (*R* = 0.924), and LooCV (*R* = 0.857), and all were highly significant. The average error was for Wild criteria (*E* = 2.17), Captive criteria (*E* = 2.23), Wild and Captive criteria (*E* = 1.30), and LooCV (*E* = 1.75). To predict the suitability of one species (LooCV) only on Wild criteria or Captivity criteria appears to be less accurate and reliable. Predicting the suitability of a new species shows an intermediate error and correlation. These data show that assessment of suitability can be based on judgment of separate criteria, that information of natural living (Wild criteria) and captive living (Captive criteria) combined give a better estimate and that new species may be added using the basic model that is developed. There is additive value in the assessments of the AS3-team, but whether the extra assessments are necessary is a matter of discussion.

**Table 6:** **Average criteria (weighted) and log odds ratio per species, predicted on base of multinomial logistic regression (see text) as the gold standard, based only on Wild or Captive criteria, based both on Wild and Captive criteria and cross-validated by leaving out one species (LooCV; *N* = 89)**.

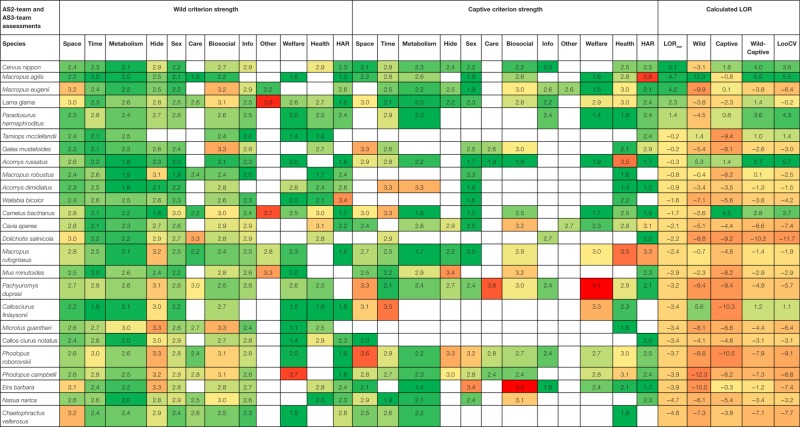

*The first 25 species are shown; the complete list in Supplementary Material (Data Sheet 1.ZIP). Colors are based on conditional formatting in MS Excel 2010. All cells are formatted per column based on a three- color gradual scale from green (related to a high suitability as pet), via yellow (related to a medium suitability as pet) to red (related to a low suitable as pet). Empty cells indicate the absence of oneliners in the database*.

The scores of the AS2-team are averaged per criteria. Correlations are calculated between pairs of criteria (Table 8). Of the 144 correlations, 54 were significant. Of special interest is the criterion animal welfare in the Captive context. This criterion is significantly (*P* < 0.01) correlated with the scores in the Wild context of Time (*R* = 0.368), Shelter (*R* = 0.179), Sex (*R* = 0.268), and Care (*R* = 0.250). There is a significant correlation between the matrices of the AS2- and the AS3-team (Mantel’s *Z* Statistic = 6.46, Pearson’s correlation *R* = 0.77, *P* < 0.001). Classifications using the ALM model and including this pattern of correlations between criteria will increase the accuracy of the model. In general, the model is expected to become better in predicting the pet suitability of a species when more species are analyzed.

### Final Order of Pet Suitability

Pet suitabilities of mammal species are first assessed by instructed animal scientists resulting in raw scores, expressed as the Logarithm of the Odds Ratio, LOR_raw_ (Table 5). Using a statistical technique – MLR – their assessments are corrected for bias between species and between assessors (LOR_cor_). Using the same techniques assessments of a limited number of stakeholders (labeled NGO1, NGO2, together NGO) and an additional independent group of animal scientists are determined (AS4). This last group appeared to be significantly different from the AS3-team concerning the range of assessments and is split in a high and low scoring group (AS4-high, AS4-low). Furthermore, a model is made that relates assessment scores on oneliner level (AS2-team) to the suitability assessment of the AS3-team (called AS2-model), while cross-validation (LooCV) also gave a pet suitability measure. The total of suitability assessments (LOR_cor_) of all analyzed species is given (Tables 5 and 7). Because all assessors used the same data from the same database to give their assessments the sequence of species, LOR_cor_ can be compared between the groups. It can be expected that the AS3-team as they are instructed to produce a strong relation with the underlying information of the database per species produces the best possible biological interpretation of the data (gold standard), leading to the assessment that five species are suitable to be kept as pets. When the assessments of both NGOs are combined no species is assessed suitable. NGO1 assessed no species and NGO2 assessed 10 species to be suitable which were mainly domesticated species. The LOR_cor_ of the 90 species assessed by the NGOs are significantly positive correlated with the LOR_cor_ of the AS3-team (AS3~NGO1, *R* = 0.578, *P* < 0.001; AS3~NGO2, *R* = 0.480, *P* < 0.001; AS3~NGOs, *R* = 0.722, *P* < 0.001). The AS4-team and the selected AS4-low group assessed no species suitable, while the AS4-high group assessed the same species suitable as the AS3-team plus an additional 7 species, totaling 12 of the 90 species analyzed (Table 7). The LOR_cor_ of the 90 species assessed by the AS4-team were significantly positive correlated with the LOR_cor_ of the AS-teams (AS3~AS4, *R* = 0.863, *P* < 0.001; AS3~AS4-low, *R* = 0.721, *P* < 0.001; AS3~AS4-high, *R* = 0.907, *P* < 0.001). By combination of assessments of the AS3-team, the NGOs, and the AS4-team judgments (ALL; Table 7), no species are assessed suitable by all assessors combined (*N* = 22). Model assessment of suitability based on the 24 criteria of behavior, welfare, health and HAR (AS2-model) showed a reasonable agreement with the AS3-team assessment (four of the five species the same as the AS3-team, while three additional species are judged suitable) (AS3~AS2-model, *R* = 0.917, *P* < 0.001). In a model to assess new additional species simulated by omitting the final judgment of the analyzed species (LooCV) almost the same classification as in the AS2-model was found, except for one species. A significant correlation between the assessment rank order of the AS3-team and the predicted LooCV rank order (Table 7) is found (AS3~LooCV, *R* = 0.854, *P* < 0.001).

**Table 7:** **Final order of pet suitabilities, based on several methods, and expressed as corrected LOR_cor_ of pet suitability**.

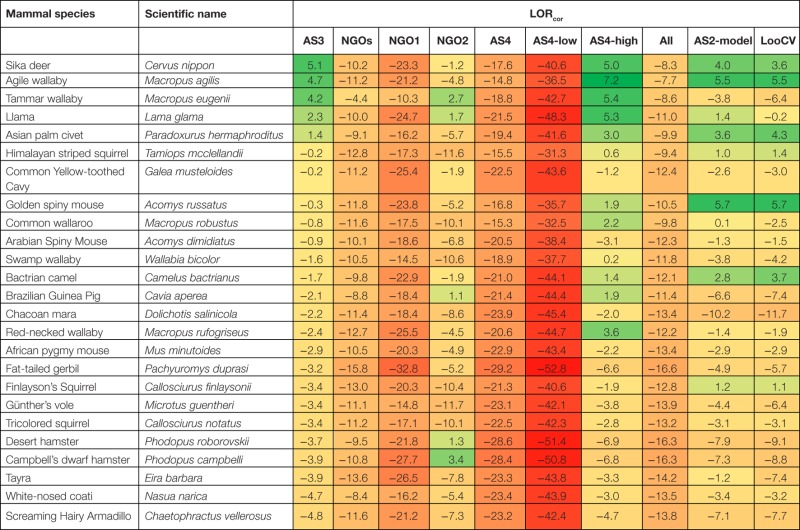

*AS3 is used as the gold standard. AS3, data from the AS3-team; NGOs, data from all stakeholders; NGO1, data from stakeholder 1; NGO2, data from stakeholder 2; AS4, data from the AS4-team; AS4-low, data from the five members from the AS4-team that assessed all mammals unsuitable as pet; AS4-high, data from the five members from the AS4-team that assessed many mammals suitable as pet; All, data of all 22 assessors combined; AS2-model, data modeled from AS2-team assessments; LooCV (leave-one-out cross-validation, data modeled based on all minus one estimation). More explanation in the text. The first 25 species are shown; the complete list is in Supplementary Material (Data Sheet 1.ZIP). Colors are based on conditional formatting in MS Excel 2010. All cells are formatted per column based on a three- color gradual scale from green (related to a high suitability as pet), via yellow (related to a medium suitability as pet) to red (related to a low suitable as pet)*.

## Discussion

An attempt was made to list mammal species that are suitable to be kept as pet. A framework is proposed in which bibliographic information of behavior ecology is used to predict species’ pet suitability. Also *in situ* observed behavior and assessed welfare may be added to the bibliographic database using the same basic structure, as is done earlier for zoo animals (39).

### Collected Oneliners

To construct a first list, concise statements – called oneliners – about the behavior, welfare, health, or HAR were collected within a restricted time frame from a limited number of sources. There were large differences in the amount of statements that were found between species and between criteria. The limited time had the advantage for species of which not much information appeared to be available, because enough time was available to search extensively for any statement concerning such a species. In case a large amount of information was available, the search was stopped after a limited amount of time (on average 4 h was spent per species) so the number of oneliners of these species was censored. It was also attempted to find as much oneliners for each of the 12 defined criteria both in the Wild and in the Captive context. Because the method is built around an initial, first or starting list together with a method to add, remove or change information, the process of determining the pet suitability of a species is designed to be dynamic and should be updated continuously, preferably after certain intervals, for instance, every year, 2-year, or 5-year period. In addition to Web of Science, we used information of encyclopedias, websites, etc. This produced sometimes identical oneliners in the database. However, by limiting the number of analyzed oneliners per sub-criterion to five, we skipped as much identical oneliners as possible making the workload and time consumption to assess lower. In future it may be possible using big data approaches to simplify and speed up the oneliner collection process; if done thoroughly it is the most time-consuming step in the pet suitability analysis proposed here. Data in criteria and subcriteria are often missing. Extra effort is needed to fill in these gaps with bibliographic information.

### Strength of Needs and Risks

Ideally, the bibliographic data for determining the suitability of a species are quantitative, for example, PanTheria for ecological analysis (74, 75). Such data are not easily found for behavioral needs and welfare risks of species. Instead, we hoped to find oneliners in which quality or quantity statements could be found about a specific behavioral need or welfare risk. In practice, this was in many oneliners not the case, which meant that the assessment depended on the knowledge and basic attitude of the assessor. Also, it was expected that differences between assessors in basic attitudes toward animals, nature, and captivity was low and had little impact on the strength of assessment (1–5) ratings. More assessors with relevant scientific background could enhance the reliability and validity of the scored needs and risks (42, 43). Also selection of the group concerning knowledge about behavior, welfare, health, and HARs could enhance the value of the scores. Of the eight animal scientists four were applied ethologists with at least 10 years’ experience in the field. The judgments of needs and risks of species (90) and criteria (24) correlated highly suggesting that the underlying data given influenced the ratings of each assessor in the same way. However, only few species are assessed suitable to be kept by owners without specialist knowledge and skills. Also, the knowledge about animal and human health issues within the teams was limited and can be ameliorated in future. The correlations found between the assessed strength of criteria in the Wild and Captive context gave a strong basis for the assumption that high demands – as specialists often display – on the environment of the animal can lead to behavior, welfare, or health risks in captivity. This is in line with the findings of Mason (5), who found a relation between home range in nature and stereotypies and juvenile mortality in captivity. On basis of this, we expected a relation between the criteria Space Wild and Welfare Captive (related to stereotypies), but such a relation is not found. Instead we found a relation between Space Wild and Health Captive (Table 8). Such relationships appeal as it is expected that species characteristics in the wild in general may influence behavior and welfare in captivity (5, 10, 13). Furthermore, the found relationships may be used for optimizing a final framework of suitability of mammal species based on the strength of oneliner scores (see Model of Suitability Based on Oneliner Strengths).

**Table 8:** **Spearman rank correlations between strength of behavioral needs or risks extracted from oneliners concerning the Wild context (rows) with needs and risks extracted from literature from the Captive context (columns)**.

*Team AS2*	*Space*	*Time*	*Metabolism*	*Shelter*	*Sex*	*Care*	*Biosocial*	*Info*	*Other*	*Welfare*	*Health*	*HAR*
*Space*	0.140	−0.115	−0.121	−0.083	0.344**	0.217*	−0.041	−0.142	−0.307	0.038	0.213**	−0.101
*Time*	0.387**	0.170*	0.067	−0.081	0.104	0.005	0.139*	0.113	0.036	0.368**	−0.051	−0.017
*Metabolism*	0.226**	−0.092	0.318**	0.436**	0.218**	0.037	0.134*	0.108	−0.162	0.096	0.169**	0.101
*Shelter*	0.465**	0.012	−0.013	0.159	0.440**	0.014	0.206**	−0.029	0.653**	0.179**	0.159**	0.108
*Sex*	0.107	−0.160*	−0.194**	0.161	0.556**	0.075	0.066	0.059	0.414*	0.268**	0.043	−0.001
*Care*	0.351**	0.157	0.280**	0.591**	0.192*	0.394**	0.178*	0.351**	0.628**	0.250**	0.183*	−0.219**
*Biosocial*	0.432**	−0.127	0.131	0.396**	0.421**	−0.037	0.158*	0.367**	0.341	0.128	0.196**	0.027
*Info*	0.092	0.033	0.072	0.113	0.226**	0.297**	0.320**	0.316**	0.450*	0.140*	0.235**	−0.019
*Other*	−0.220	−0.371**	−0.029	0.085	0.256*	0.695**	−0.217	0.390**	0.625**	0.202	0.042	−0.529**
*Welfare*	0.517**	0.068	0.024	0.134	0.296**	0.163	0.106	0.125	0.117	0.223**	0.224**	0.084
*Health*	0.148	0.119	0.250**	−0.008	0.162*	−0.005	0.372**	0.148	0.288	−0.094	0.424**	0.105
*HAR*	0.358**	−0.269**	0.416**	0.221*	0.216**	0.497**	0.579**	0.411**	0.270	0.361**	0.091	0.368**

For example, high Time needs in the Wild context are significantly positive related with high Welfare risks in the Captive context (*R* = 0.368). Significant Rho = bold, italic; *P < 0.05; **P < 0.01.

### Assessing Pet Suitability

The data show a wide range of judgments about *pet suitability* in the animal scientists (AS3, AS4, and the split AS4-high and AS4-low group). The instructed assessment of suitabilities of mammal species, in which assessors are urged to base their assessment on firm arguments worked, because a strong relation between suitability and assessments of criteria was found. Differences between assessors and assessments of species were controlled for by MLR making the suitability assessment more objective and less discriminatory. Uninstructed assessors and assessment appeared to produce same or at least comparable orders of suitabilities. However, the decision or cut point between suitability and unsuitability was markedly different. This was best shown by the AS4-team assessments that could be split into a group that judged all species as unsuitable to be kept as pet (AS4-low) and a group that judged 12 species suitable to be kept as pet (AS4-high), which was more than the number that the instructed group (AS3) judged suitable. It is to be expected that in case more assessors are involved in the suitability assessment and the basic attitude and/or stakeholder associations are clear a more complete view about pet suitability could be given. A large group of instructed assessors is necessary for a firm baseline. In addition, more oneliners of all predefined criteria also will enhance the accuracy of the suitability judgments. Still, in the end even with large numbers of assessors the combination of many subjective assessments is still not an objective assessment, but probably as objective as possible and reflecting the actual scientific opinion as good as possible. In future, decisions on pet suitability may be based on a quantitative approach using data from research as for instance is done in the ecological database PanTheria (74–78). Until that level of detail is reached, a qualitative database with additional assessments of experts is the best approach possible.

The correction of raw scores for assessor and species differences has effects on the final list of suitable mammal species. However, this correction is necessary according to the Andibel judgment.^2^

### Pet Suitability Framework and Oneliners

In the framework as presented here, selection and assessment of information play a significant role. Selection by the AS1-team (oneliners), assessment by the AS2-team (strength of needs and risks), assessment by the AS3-team (suitability), and assessments, for instance, by stakeholders are needed to formulate a final rank order of pet suitabilities. A definitive cut-off point (threshold) above which mammals are suitable and under which mammals are judged unsuitable could not be given. Such a threshold is largely dependent on state of the art in science, societal influences, culture, etc. In this paper, a best professional judgment is given by the AS3-team that was instructed in detail to base their assessments on the data provided. The cutpoint or threshold of this group is the best professional judgment we have for the time being. Two steps could be made more efficient in the current framework, especially concerning analysis of more species. Since no quantitative database of mammal species characteristics – as suggested in the preceding paragraphs – exists, the activities of the AS1-team and AS2-team could not be skipped. The activities of the AS3-team could be firmly reduced because – as the AS3-team is instructed – a strong relation between the strength of needs and risks is found in determining the eventual pet suitability. This relation may act as an assessment model for additional species and may be updated after certain intervals. The rank order of suitabilities produced by the different teams and the classifications made of the rank order shows that as an initial estimate the framework produces rank orders that correlate strongly and may be used in practice. Determining the threshold above which species have a sufficient high pet suitability (LOR_cor_) and are consequently suitable to be kept as pet cannot be determined objectively, unless actual behavior, welfare, and health data of species become available.

### Final Order and Future of Pet Suitability

Based on the bibliographic data found and included in the objective and non-discriminatory framework the following mammal species are judged suitable to be kept as pet, i.e., *Cervus nippon, Macropus agilis, Macropus eugenii, Lama glama*, *and Paradoxurus hermaphroditus* (AS3-team). Based on more strict assessments no mammal species could be kept (AS4-low team) or less strict assessment *Macropus rufogriseus, Macropus robustus, Cavia aperea, Acomys russatus, Camelus bactrianus, Tamiops mcclellandii*, and *Wallabia bicolor* may be added (AS4-high team). The NGOs that participated with only few assessors showed that domesticated species have a relative high chance of being suitable (NGO2: 4 out of 10 were domesticated^3^ and three species were semi-domesticated).^12^ Based on some models comparable classifications of mammal species were found.

In the course of time, the framework was simplified by excluding a number of planned elements. It is possible to include these elements in future developments of the framework. The accuracy of the framework will increase when time limitations in searching, selection, and assessment of oneliners are removed, the quality of the bibliographic source is added, and data on housing and management conditions are included. The use of indicator species (guide species of flagship species that represents a collection of related and comparable species) was originally developed and could be included again and developed further especially for genera and groups with a very large number of species, such as the shrews (*Soricidae*).

Although high needs of different criteria or risks may be differently related to pet suitability of a species, no weighing of strength of criteria is done in the current framework. The correlations between strength of needs and risks show that some input criteria might be differentially related to welfare, health, and HAR. For instance, high scores on Space needs may in the end appear much more important than high scores on Information needs. Thus, weighing of assessments between criteria could also increase the accuracy of the framework. The framework also shows which data of behavioral ecology are lacking for specific species. Additional data on behavior, welfare, health, and HAR of species can be included in the framework as is done for zoo mammals (39) or included after being published. In this way, the pet suitability framework also stimulates and directs applied animal welfare research. Participation of stakeholders could enhance the available bibliographic information and add to the framework by showing and integrating their viewpoints. Therefore, no polarized discussion but dialog between stakeholders – and researchers – will improve the assessment of pet suitability (79, 80).

The framework we developed may also be relevant not only for the Netherlands but other countries may also be interested using it in developing housing requirements for certain species. Cooperation and validation of the framework should have a high priority. The assessments within the various teams were correlated so the internal validity was measured, but external validation is lacking. In a first attempt for external validation, we invented an additional method called the Pet Exaptation Index (81) based on species characteristics favorable for domestication (82). External validation needs attention in the near future and can be based on practical experiences of vets or rescues and may be supported by *in situ* observations of mammal species kept as pets.

In conclusion, a framework is proposed to assess the pet suitability of mammal species based on bibliographic knowledge of their behavioral ecology. An initial list of 90 species is made by rank ordering species on base of estimated pet suitability. The framework is dynamic and has the potential to increase the objectivity of decisions on pet suitability of mammal and other animal species.

## Author Contributions

PK led the project, designed the study, collected additional data, analyzed the data, and drafted the manuscript; RM participated in the design of the study, managed the database, and commented on the manuscript; BI managed the project, participated in the design of the study, and commented on the manuscript. All authors approved the final manuscript.

## Conflict of Interest Statement

The author declares that the research was conducted in the absence of any commercial or financial relationships that could be construed as a potential conflict of interest.

## Footnotes

^1^*Five Freedoms*. (2015). Available from: http://webarchive.nationalarchives.gov.uk/20121007104210/http:/www.fawc.org.uk/freedoms.htm

^2^*Judgement of the Court*. (2015). Available from: http://eur-lex.europa.eu/legal-content/EN/ALL/?uri=CELEX:62007CJ0219

^3^*List of Domesticated Animals*. (2012). Available from: https://en.wikipedia.org/wiki/List_of_domesticated_animals

^4^*ARKive Online*. (2013). Available from: http://www.arkive.org/

^5^*Mammalian Species*. (2013). Available from: http://www.science.smith.edu/msi/

^6^*The IUCN Red List of Threatened Species*. *Version 2012*. (2013). Available from: http://www.iucnredlist.org

^7^*The Encyclopedia of Life*. (2013). Available from: http://www.eol.org

^8^*Wikipedia Nederlands*. (2013). Available from: http://nl.wikipedia.org/

^9^*The Free Encyclopedia*. (2013). Available from: http://en.wikipedia.org/

^10^*Animal Diversity Web*. (2013). Available from: http://animaldiversity.ummz.umich.edu/site/accounts/information/Mammalia.html

^11^i.e., the DIBEVO (trade association for entrepreneurs in the entire pet industry in the Netherlands), AAP Foundation (AAP is committed to creating a better future for primates and other exotic mammals), NBvH (Dutch Hobby and Smallholders Association), Animal Coalition (Cooperating Animal Welfare Organizations Netherlands), PVH (Platform for Responsible Pet Ownership), Association of Park Animal Owners, Association of In- and Exporters of Birds and Hobby Animals, Dierenbescherming (Dutch Society for the Protection of Animals), and KNMvD (Royal Dutch Society for Veterinary Medicine).

^12^*List of Domesticated Species*. (2015). Available from: https://en.wikipedia.org/wiki/List_of_domesticated_animals
